# The Effects of Tocotrienol on Bone Peptides in a Rat Model of Osteoporosis Induced by Metabolic Syndrome: The Possible Communication between Bone Cells

**DOI:** 10.3390/ijerph16183313

**Published:** 2019-09-09

**Authors:** Sok Kuan Wong, Kok-Yong Chin, Soelaiman Ima-Nirwana

**Affiliations:** Department of Pharmacology, Faculty of Medicine, Universiti Kebangsaan Malaysia, Jalan Yaacob Latif, Bandar Tun Razak, Cheras 56000, Kuala Lumpur, Malaysia (S.K.W.) (K.-Y.C.)

**Keywords:** osteoblast, osteoclast, osteocyte, osteoporosis, vitamin E

## Abstract

A positive association between metabolic syndrome (MetS) and osteoporosis has been demonstrated in previous animal studies. The mechanisms of MetS in orchestrating the bone remodelling process have traditionally focused on the interactions between mature osteoblasts and osteoclasts, while the role of osteocytes is unexplored. Our earlier studies demonstrated the bone-promoting effects of tocotrienol using a rat model of osteoporosis induced by MetS. This study aimed to investigate the expression of osteocyte-derived peptides in the bone of rats with MetS-induced osteoporosis treated with tocotrienol. Age-matched male Wistar rats (12-week-old; *n* = 42) were divided into seven experimental groups. Two groups served as the baseline and normal group, respectively. The other five groups were fed with a high-carbohydrate high-fat (HCHF) diet to induce MetS. The five groups of HCHF animals were treated with tocopherol-stripped corn oil (vehicle), annatto tocotrienol (60 and 100 mg/kg), and palm tocotrienol (60 and 100 mg/kg) starting from week 8. At the end of the study, the rats were sacrificed and their right tibias were harvested. Protein was extracted from the metaphyseal region of the proximal right tibia and levels of bone peptides, including osteoprotegerin (OPG), soluble receptor activator of nuclear factor-kappa B ligand (sRANKL), sclerostin (SOST), Dickkopf-related protein 1 (DKK-1), fibroblast growth factor-23 (FGF-23), and parathyroid hormone (PTH), were measured. The vehicle-treated animals displayed higher levels of sRANKL, SOST, DKK-1, FGF-23, and PTH as compared to the normal animals. Oral supplementation of annatto and palm tocotrienol (60 and 100 mg/kg) reduced the levels of sRANKL and FGF-23 in the HCHF animals. Only 100 mg/kg annatto and palm tocotrienol lowered SOST and DKK-1 levels in the HCHF animals. In conclusion, tocotrienol exerts potential skeletal-promoting benefit by modulating the levels of osteocytes-derived bone-related peptides.

## 1. Introduction

The occurrence of three out of five medical abnormalities (obesity, hyperglycaemia, hypertension, hypertriglyceridemia and low high-density lipoprotein (HDL) cholesterol), also termed as metabolic syndrome (MetS), might predispose an individual to skeletal disturbance [1]. Our earlier studies demonstrated that the rats fed with high-carbohydrate high-fat (HCHF) diet fully developed MetS after 12 weeks, characterized by the fulfilment of all criteria of MetS (increased abdominal circumference, blood glucose, blood pressure, triglycerides, and lowered HDL cholesterol) [2]. Significant bone loss was also detected subsequent to the establishment of MetS, with the alteration of bone histomorphometric parameters being characterized by increased osteoclastic activity and reduced osteoblastic activities. The imbalance between bone formation and resorption processes favoured deterioration of bone microstructure and bone strength [3,4]. The maintenance of bone integrity relies on the balance between the tightly-coupled bone formation and resorption, being mainly governed by osteoblasts and osteoclasts. The role of osteocytes in MetS-mediated changes in bone metabolism receives far less attention when compared to osteoblasts and osteoclasts.

Osteocytes are the most abundant bone cells that are differentiated from osteoblasts and permanently residing within the mineralized bone matrix. They act as mechanosensors, communicators and coordinators in maintaining bone quality. Osteocytes detect mechanical and hormonal stimuli as well as transmitting the osteocyte-secreted molecules through the lacunar-canalicular network, allowing for communication with other bone cells to regulate bone resorption and formation [5]. Osteocytes produce most of the molecular mediators that are produced by osteoblasts, with osteocytes being the more dominant source than osteoblasts [6]. Osteocytes play a critical role in the regulation of osteoblast activity, osteoclast activity, and calcium homeostasis. They regulate bone formation through wingless (Wnt)/β-catenin signalling, a crucial pathway that directs the commitment of mesenchymal stem cells into osteoblastic lineage. Osteocytes secrete sclerostin (SOST) and Dickkopf-related protein 1 (DKK-1), which negatively regulate the activation of Wnt/β-catenin by preventing the binding of Wnt ligands to the frizzled receptor, lipoprotein receptor-related protein (LRP) 5 and LRP6 [7]. The differentiation of osteoclast precursors into mature osteoclasts is mediated by osteoprotegerin (OPG) and the receptor activator of nuclear factor-kappa B ligand (RANKL) produced by osteocytes. The increased expression of RANKL facilitates osteoclastogenesis [8]. On the other hand, OPG inhibits osteoclastogenesis by binding to RANKL, thus preventing the receptor activator of nuclear factor-kappa Β (RANK)-RANKL interaction. Fibroblast growth factor-23 (FGF-23) is also the products of osteocytes, whereas osteocytes are the critical effectors for parathyroid hormone (PTH) action. They are both implicated in the regulation of phosphate homeostasis and vitamin D metabolism, which reiterates their role in the maintenance of calcium homeostasis [9,10,11]. 

In view of the role of osteocytes in the synthesis of molecules essential for bone development, targeting the osteocyte-driven skeletal remodelling could be the next step in developing anti-osteoporotic agents. Several studies suggested that vitamin E could influence bone metabolism [12]. Vitamin E can be categorized into two major groups: tocotrienol and tocopherol. Each of these groups consist of four different isomers that are based on the location of methyl groups on the chromanol nucleus, namely alpha- (α-), beta- (β-), gamma- (γ-), and delta-(δ-)tocotrienol. Palm fruit and annatto seed are some of the abundant sources of tocotrienol. Palm-based tocotrienol contains approximately 75% tocotrienol and 25% tocopherol, whereas annatto-derived tocotrienol solely consists of tocotrienol (99.9%) with the absence of tocopherol [13]. The ability of tocotrienol mixture to mitigate MetS-associated medical conditions [14] and bone deterioration [15,16] has been well-established. Tocotrienol was found to alleviate MetS features through its potent anti-inflammatory [17], anti-oxidative [18,19] and cholesterol-reduction properties [20]. Besides, tocotrienol potentially mediates bone metabolism through regulating the release of inflammatory mediators, reactive oxygen species (ROS), growth factors, and hormones [12]. Interestingly, we found that the oral administration of annatto and palm tocotrienol conferred beneficial effects in reversing MetS features and preventing bone loss that is caused by MetS [21,22,23]. 

The effects of vitamin E on osteocytes have been reported by several researchers. A study by Jia et al. (2016) demonstrated the inhibitory effects of vitamin E (content not mentioned) on osteocyte apoptosis and oxidative damage using a rabbit model of osteonecrosis induced by steroid [24]. Another study by Hagan et al. (2019) indicated that the healthy mice fed with vitamin E-deficient diet had a higher percentage of wounded osteocytes but lower periosteal mineralizing surface, mineral apposition rate and bone formation rate when compared to the mice that were fed with regular diet [25]. In the same study, found that necrosis of MLO-Y4 osteocytes caused by hydrogen peroxide was prevented by vitamin E (α-tocopherol and Trolox) pre-treatment [25]. Another recent in vitro study indicated the anti-oxidative effects of palm-derived δ-tocotrienol, protecting the MC3T3-E1 osteoblasts and MLO-Y4 osteocytes against oxidative damage that is induced by *tert*-butyl hydroperoxide [26]. These studies mainly focused on the effects of vitamin E on osteocyte viability through its capability of alleviating oxidative damage.

This present study aimed to assess the role of osteocytes in regulating bone remodelling in an animal model of bone loss that is induced by MetS. A panel of osteocyte-related peptides, including OPG, RANKL, SOST, DKK-1, FGF-23, and PTH, was evaluated in this study. 

## 2. Materials and Methods 

### 2.1. Treatments

Annatto tocotrienol that was derived from *Bixa orellana* containing 16% γ-tocotrienol and 84% δ-tocotrienol was a gift from American River Nutrition Inc. (Hadley, MA, USA) (Lot Number: 5Dl3-AMK2-70). Palm tocotrienol extracted from *Elaeis guineensis* containing 24.7% α-tocotrienol, 4.5% β-tocotrienol, 36.9% γ-tocotrienol, 12.0% δ-tocotrienol, and 21.9% α-tocopherol was a gift from Excelvite Sdn. Bhd. (Chemor, Malaysia) (Lot Number: A1/50/0159_1_120315). Annatto and palm tocotrienol were diluted with tocopherol-stripped corn oil (MP Biomedicals, Solon, OH) prior to administration.

### 2.2. Animal Experimentation

The study protocol was reviewed and approved by Universiti Kebangsaan Malaysia Animal Ethics Committee (UKMAEC) (Approval Number: PP/FAR/2015/IMA/20-MAY/679-JUNE-2015-MAY-2017). Twelve-week-old male Wistar rats (*n* = 42) were purchased from Laboratory Animal Resource Unit (LARU), Universiti Kebangsaan Malaysia (Kuala Lumpur, Malaysia). The animals were singly housed in the Animal Facility of the Department of Anatomy, Faculty of Medicine, Universiti Kebangsaan Malaysia (Cheras, Malaysia) with the ambient temperature of 25 ± 2 °C and alternated 12 hours light-dark cycle. Food and water were provided *ad libitum*. Following acclimatization for one week, the animals were randomly assigned into seven experimental groups (*n* = 6/group). The baseline group was sacrificed after acclimatization. The normal group was fed with standard rat chow and tap water. The remaining five groups were given a high-carbohydrate high-fat (HCHF) diet with 25% fructose-supplemented drinking water to induce MetS [26]. The HCHF diet was prepared using 395 g sweetened condensed milk, 200 g ghee, 175 g fructose, 155 g powdered rat food, 25 g Hubble Mendel Wakeman salt mixture, and 50 mL water. The treatments were commenced at week 8 and given on a daily basis for 12 weeks. The normal animals were orally administrated with normal saline. The HCHF animals were orally treated with tocopherol-stripped corn oil (vehicle), 60 mg/kg annatto tocotrienol, 100 mg/kg annatto tocotrienol, 60 mg/kg palm tocotrienol, and 100 mg/kg palm tocotrienol. At week 20, the animals were sacrificed and right tibias were harvested for the measurement of bone-related peptides.

### 2.3. Protein Extraction and Quantification

**Figure 1:**
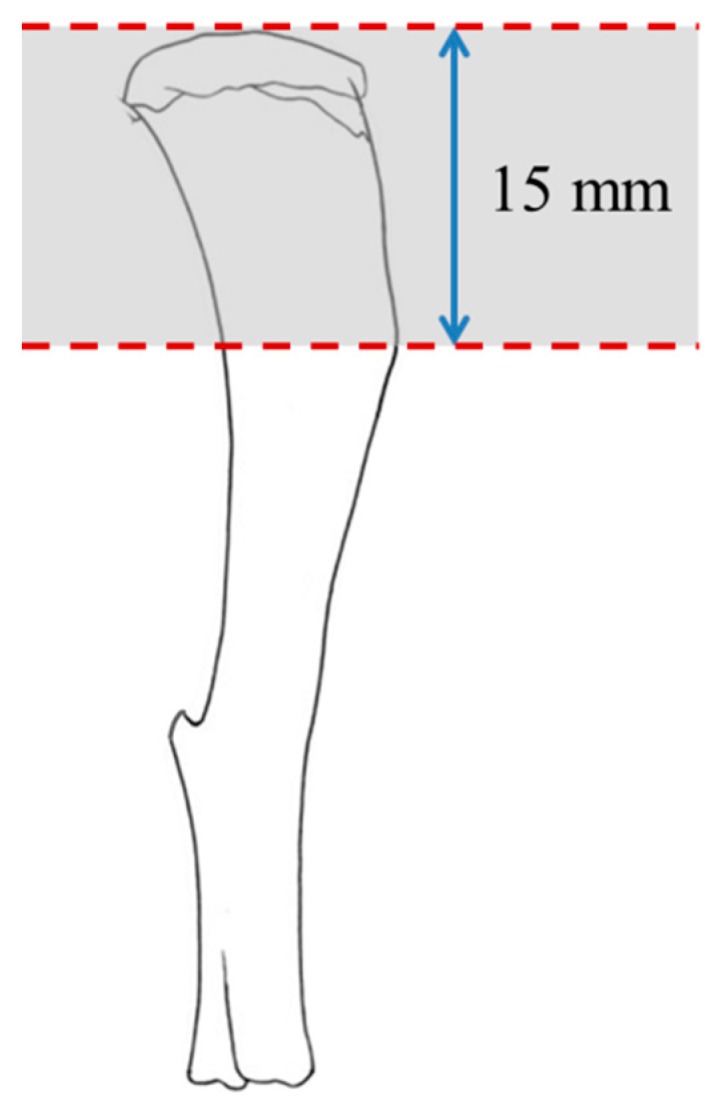
The metaphyseal region of tibia chosen for analysis.

Right tibias that were excised from the animals were cleaned of soft tissues and weighed. A transverse cut at the metaphysical region of the proximal right tibia (15 mm from the proximal end of the tibia) (250–270 mg) was made (Figure 1). This region was selected, as our previous study detected a deterioration of trabecular bone microstructure in this animal model of bone loss induced by MetS [4]. Briefly, the excised bone section (both cortical and cancellous bone without fibula) was immersed with extraction buffer (containing 50 mM Tris (pH 7.4), 150 mM sodium chloride (NaCl), 1% Triton X-100, 1% sodium deoxycholate (C_24_H_39_O_4_Na), 1 mM ethylenediaminetetraacetic acid (EDTA), 0.1% *sodium dodecyl* sulphate (SDS), 10 nM sodium fluoride (NaF), 1 mM sodium orthovanadate (Na_3_VO_4_), and 1 mM phenylmethylsulfonyl fluoride (PMSF)) (Elabscience Biotechnology Inc., China) in bead ruptor tubes pre-filled with metal bead. The samples were homogenised using a high-speed homogenizer (OMNI Bead Ruptor 24, Kennesaw, GA) at the speed of 6.95 m/s and cycle time of 8 × 45 s with 45 s dwells, followed by centrifugation at 8000 g for 10 minutes. Supernatant was collected and protein quantification of all bone samples was performed using Bradford method (Quick Start^TM^ Bradford Protein Assay, Bio-Rad Laboratories Inc., CA, USA).

### 2.4. Assessment of Bone-Related Peptides

Levels of OPG, SOST, DKK-1, FGF-23, and PTH in the metaphyseal region of the proximal tibia were quantified using Milliplex^®^ Map Rat Bone Magnetic Bead Panel (Catalogue Number: RBN1MAG-31K, EMD Millipore Corporation, Billerica, USA) and measured using Luminex 100 (Multiplexed Biomarker Immunoassays for Luminex^®^ Instrumentation/xMAP^®^ Technology-Luminex Corporation, Austin, USA). The level of soluble receptor activator of nuclear factor-kappa B ligand (sRANKL) was determined using enzyme-linked immunosorbent assay kit, according to the manufacturer’s instructions (Catalogue Number: ER1604, FineTest, Wuhan, China). The levels of bone-related peptides were normalised with the protein concentration of each sample.

### 2.5. Statistical Analysis

Statistical Package for Social Sciences (SPSS) version 20 (IBM, Armonk, USA) was used to perform statistical analysis. All the data were normally distributed. Statistical differences between groups were assessed by one-way analysis of variance (ANOVA) with *Tukey’s* post-hoc pairwise comparison test. All of the results were presented as mean ± standard error of the mean (SEM). *P* values of <0.05 were considered to be statistically significant. 

## 3. Results

**Figure 2:**
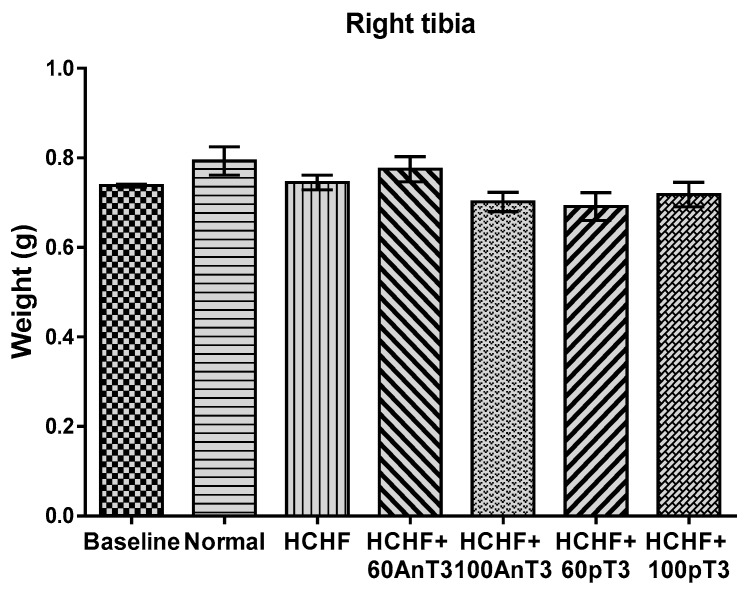
The weight of right tibia in the baseline, normal, high-carbohydrate high-fat (HCHF) animals with and without treatment of annatto and palm tocotrienol (60 and 100 mg/kg). Data are presented as mean ± SEM.

The weight of right tibia did not show any significant difference between all of the experimental groups (*p* = 0.059) (Figure 2).

**Figure 3:**
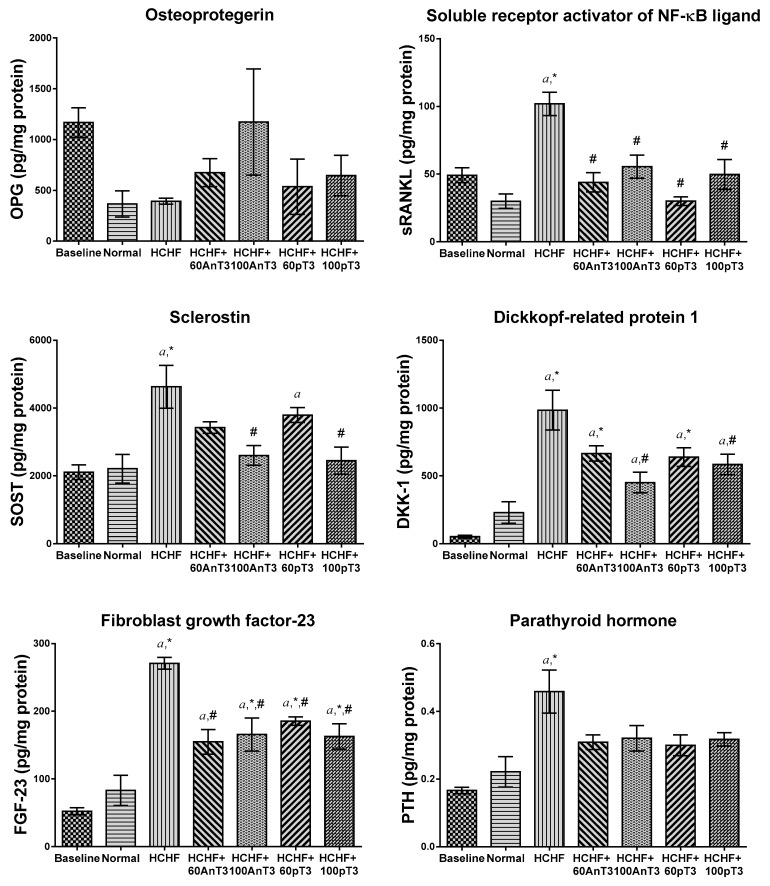
Level of osteoprotegerin (OPG), soluble receptor activator of nuclear factor-kappa B ligand (sRANKL), sclerostin (SOST), Dickkopf-related protein 1 (DKK-1), fibroblast growth factor-23 (FGF-23), and parathyroid hormone (PTH) in the baseline, normal, HCHF animals with and without treatment of annatto and palm tocotrienol (60 and 100 mg/kg). Data are presented as mean ± SEM. Letter ‘*a*’ indicates a significant difference (*p* < 0.05) compared to the baseline group, symbol ‘*’ indicates a significant difference (*p* < 0.05) compared to the normal group and ‘#’ indicates a significant difference (*p* < 0.05) compared to the HCHF group.

The assessment of bone-related peptides indicated that HCHF diet significantly raised the protein levels of sRANKL (*p* < 0.001), SOST (*p* = 0.001), DKK-1 (*p* < 0.001), FGF-23 (*p* < 0.001) and PTH (*p* = 0.001) as compared to the normal rats that were fed with standard diet. Supplementation of annatto and palm tocotrienol at the dose of 60 and 100 mg/kg reduced the levels sRANKL (60AnT3: *p* < 0.001; 100AnT3: *p* = 0.002; 60pT3: *p* < 0.001; 100pT3: *p* < 0.001) and FGF-23 (60AnT3: *p* < 0.001; 100AnT3: *p* = 0.001; 60pT3: *p* = 0.014; 100pT3: *p* = 0.001) relative to the vehicle-treated HCHF animals. Only 100 mg/kg annatto and palm tocotrienol prevented the increase of SOST (100AnT3: *p* = 0.003; 100pT3: *p* = 0.003) and DKK-1 (100AnT3: *p* = 0.001; 100pT3: *p* = 0.023) levels in the HCHF animals as compared to the negative controls (Figure 3). 

## 4. Discussion

In this study, we investigated the role of osteocyte in governing the process of bone metabolism using an osteoporotic rat model induced by MetS. The effects of annatto and palm tocotrienol supplementation on the osteocyte-derived secretory proteins (OPG, sRANKL, SOST, DKK-1, FGF-23, and PTH) have been evaluated. 

The RANK/RANKL/OPG system plays a central role in osteoblast-dependent osteoclastogenesis. Osteoblasts produce RANKL in response to local bone resorption-stimulating factors, such as inflammatory cytokines, hormones, and growth factors. RANKL binds to RANK on the osteoclast precursors to activate downstream signalling molecules (nuclear factor-kappa B (NF-κB), Fos proto-oncogene (c-Fos), and nuclear factor of activated T-cells cytoplasmic 1 (NFATc1)) for the completion of osteoclast differentiation process [27]. A previous study demonstrated the crucial role of osteocytes in the production of RANKL [28]. The authors reported that purified osteocytes expressed higher amount of RANKL than osteoblasts and bone marrow stromal cells in vitro, which suggested that osteocytes are the major source of RANKL; thus, have a greater capacity to regulate osteoclastogenesis [28]. The level of RANKL in osteocytes is tightly controlled by mechanical and hormonal stimuli. In vivo study indicated that mice lacking RANKL in osteocytes were protected from bone loss due to unloading [29]. In addition, PTH acts as a potent endocrine regulator for the expression of RANKL. A transgenic mice expressing constitutively active PTH receptor in osteocytes increased RANKL expression and bone remodelling rate [30]. On the contrary, conditional deletion of the PTH receptor from osteocytes resulted in decreased bone resorption and increased bone mass [31].

A decoy receptor for RANKL, which is known as OPG, is also produced by osteoblasts and osteocytes to prevent the RANKL-RANK interaction, thereby inhibiting osteoclastogenesis [6]. Theoretically, the upregulation of RANKL expression by inflammatory mediators, hormones, and growth factors is accompanied by a lower expression of OPG in osteoblasts and osteocytes, which results in osteoclastogenesis [32]. OPG is also regulated by the canonical Wnt/β-catenin pathway. Without Wnt signal, β-catenin is readily degraded by a β-catenin destruction complex consisting of axin, adenomatous polyposis coli (APC), glycogen synthase kinase 3 (GSK3), and casein kinase 1 (CK1). The presence of Wnt signal binds to the receptor complex of frizzled, LRP5, and LRP6 causing the disruption of functional β-catenin destruction complex, which eventually leads to cytosolic accumulation and nuclear translocation of β-catenin. Subsequently, β-catenin binds to T-cell factor/lymphoid enhancing factor (Tcf/Lef) to initiate the transcription of OPG [33]. A previous *in vivo* study supported that osteocyte-specific β-catenin-deficient mice were osteoporotic due to the downregulation of OPG expression and increased osteoclast number and activity. However, the osteoblast function and osteocyte density of the mice were normal [34]. Apart from that, it has been proposed that PTH influences the expression of OPG. A study by Fu et al. (2002) indicated that PTH potently suppressed OPG expression in murine osteoblastic cells [35]. 

The canonical Wnt/β-catenin pathway is governed through two important extracellular inhibitors, SOST and DKK-1. They are osteocyte-derived Wnt antagonists that antagonize the activation of Wnt/β-catenin signalling by competitively binding to the LRP5 and LRP6 co-receptors [36]. Apart from being a negative modulator of Wnt/β-catenin pathway, SOST is also a bone morphogenetic protein (BMP) antagonist that prevents the binding of BMPs to their receptors, suppresses BMP signalling and reduces the mineralization of osteoblastic cells [37]. Many preclinical studies provided evidence that the overexpression of SOST or DKK-1 in mice resulted in reduced bone mass [37,38,39,40,41], whereas mice with the deficiency of SOST and DKK-1 gene displayed increased bone formation and higher bone mass [42,43,44]. Mechanical loading and PTH were found to negatively regulate the levels of SOST and DKK-1 expression [38,45,46,47]. Hence, the activation of Wnt signalling not only stimulates osteoblast activity, but it also attenuates osteoclast differentiation and function by stimulating the production of OPG. 

Fibroblast growth factor-23 is a protein that is primarily secreted by osteocytes and osteoblasts [48]. It is a known regulator responsible for phosphate homeostasis and vitamin D metabolism, thus having a pivotal role in the pathophysiology of bone disorders. FGF-23 suppresses the expression of sodium-phosphate co-transporter gene (NPT2) in the proximal renal tubule, thus decreasing reabsorption and increasing the urinary excretion of phosphate [10]. In addition, FGF-23 inhibits 1α-hydroxylase, thereby diminishing the conversion of 25-hydroxyvitamin D [25(OH)D] to active 1,25-dihydroxyvitamin D [1,25(OH)_2_D] and impairing calcium absorption [11]. Therefore, a high level of FGF-23 is associated with the inhibition of bone mineralization. Under physiological condition, the recognition of FGF-23 by FGF cell-surface receptors (FGFRs) requires the presence of Klotho, which is a transmembrane protein that acts as cofactor facilitating the binding and activation of FGFRs by FGF-23 [49]. Osteoblastic MC3T3-E1 cells exogenously supplemented with FGF-23 and Klotho showed reduced mineralization and osteoblast differentiation markers, including alkaline phosphatase (ALP), osteocalcin (OCN), collagen type 1 alpha 1 (COL1α1), bone sialoprotein (BSP), and osteopontin (OPN) [50]. 

Parathyroid hormone mainly acts on three major organs (kidney, intestine, and bone) to regulate the extracellular calcium and phosphate homeostasis [9], thus suggesting its role in orchestrating bone metabolism. In bone, apart from osteoblasts and chondrocytes, increasing evidence demonstrates that osteocytes are a crucial cellular target of PTH. The interaction between PTH and its membrane receptor, which is known as PTH/PTH-related protein type 1 receptor (PTHR1), results in both anabolic and catabolic skeletal responses. As aforementioned, PTH exhibited osteogenic action through the downregulation of SOST and DKK-1 (potent inhibitors of Wnt signalling), leading to increased bone formation [51]. In contrast, PTH induces osteoclastogenesis via differential regulation of OPG and RANKL, whereby OPG expression is downregulated, but RANKL expression is upregulated [52]. However, the expression of OPG might be unaffected, as it is likely counteracted by increased OPG expression resulting from Wnt activation [51]. The effects of PTH on bone, either anabolic or catabolic, depend on the duration and periodicity of PTH exposure. The transient exposure to PTH leads to enhanced bone formation, whereas sustained exposure to PTH leads to increased bone resorption [53,54].

In this current study, we observed the increase in sRANKL, SOST, DKK-1, and FGF-23 levels in the bones of the animals after being challenged with HCHF diet, whereby it might be due to the increase in the PTH level. The findings were supported by a previous study indicating that high-fat diet-induced hyperlipidaemia caused hyperparathyroidism and impaired bone regeneration and mechanical strength in mice [55]. The results from a previous human study also showed that there was a positive association between the risk of MetS and PTH level [56]. However, evidence on the effects of diet-induced MetS on the levels of other bone peptides (OPG, sRANKL, SOST, DKK-1 and FGF-23) is limited. The increase in cytokines [including interleukin-6 (IL-6) and interleukin-1 alpha (IL-1α)] was also detected in the animals that were treated with HCHF diet [21,23], possibly contributing to the increase of sRANKL in bone. There was no significant change in the level of OPG among all of the experimental groups in our present study. The possible explanation could be the direct action of PTH and inflammatory mediators in downregulating OPG expression was counteracted by the indirect action of PTH in downregulating SOST and DKK-1 expression, leading to the activation of Wnt/β-catenin and upregulation of OPG expression. Mechanical loading and unloading might not be the factors that account for the alteration in the level of these bone-related peptides (sRANKL, SOST and DKK-1) in this study because no change in the body weight of animals among all of the experimental groups was observed [21,23]. Overall, our findings demonstrated that MetS increased the levels of sRANKL, SOST, DKK-1, FGF-23, and PTH at the osteocyte level, translating into the dysregulation of the bone formation and resorption in the animals. 

The osteoprotective effects of tocotrienol were mediated through the normalization of these osteocyte-derived products. Annatto and palm tocotrienol at the dose of 60 and 100 mg/kg reduced the elevated sRANKL and FGF-23 in animals caused by long-term feeding of HCHF diet. The findings were in line with previous in vitro study that 50 μM of α-tocotrienol suppressed RANKL expression in osteoblasts and RANKL-induced expression of c-Fos and NFATc1 by inhibiting extracellular-signal-regulated kinase (ERK) and NF-κB activation [57]. Another study also revealed that Trolox (a water-soluble α-tocopherol) suppressed the IL-10-induced RANKL expression in the co-culture of osteoblasts and bone marrow cells [58]. The effects of tocotrienol treatment on FGF-23 level was previously evaluated by Ibrahim et al. (2015) using ovariectomized female rats with fracture. In that study, their results indicated that injection of tocotrienol particles for four weeks did not cause any change in the FGF-23 gene expression [59]. The distinct outcome observed between the current study and their study might be due to the variations of study design in terms of the treatment regimen, route of administration, treatment period, and type of animal model. The level of OPG was unaffected after the treatment of annatto and palm tocotrienol in the present study. Per the current findings, previous in vitro [57] and in vivo [60] studies have reported that tocotrienol did not alter OPG expression by osteoblasts. Higher doses of annatto and palm tocotrienol conferred better outcomes, whereby the increase in SOST and DKK-1 levels in the animals with MetS was prevented. A comparable study on the effects of tocotrienol on these parameters is limited. For PTH, treatment with tocotrienol did not cause any change in the HCHF diet-fed animals. We hypothesize that a longer period of treatment might be needed to reverse the elevated PTH level in the HCHF animals. In congruent with the current findings, a study by Norazlina et al. (2004) found that rats fed with vitamin E-deficient diet had a higher level of PTH, but the supplementation of vitamin E (α-tocotrienol and α-tocopherol) for three months did not cause any change in the PTH level [61]. 

**Figure 4:**
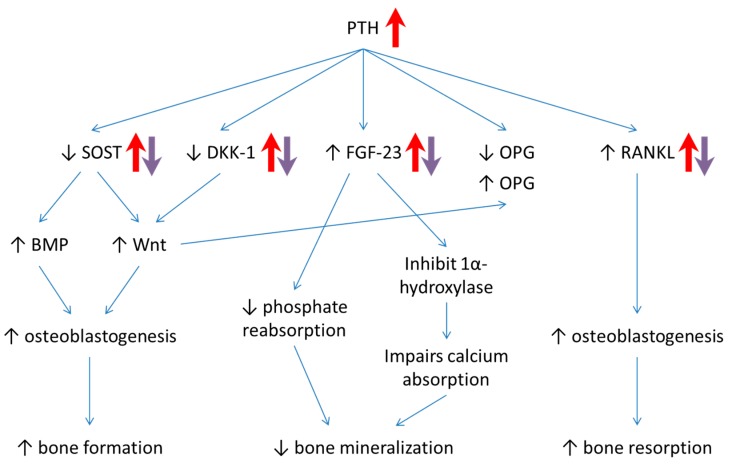
The postulated role of osteocyte-derived bone peptides (OPG, sRANKL, SOST, DKK-1, FGF-23, and PTH) in regulating bone remodelling processes. The red arrows indicate the protein levels in rats after challenged with HCHF diet for 20 weeks. The purple arrows indicate the protein levels in rats in response to the treatment with tocotrienol.

A schematic diagram that summarizes the postulated actions of osteocyte-derived molecules in MetS-induced bone loss and the effects of tocotrienol in osteocyte-driven bone remodelling is shown in Figure 4. Several limitations need to be addressed in this study. Firstly, the measurement of bone peptides was only performed at the end of the study, so we cannot monitor the sequential changes in their level throughout the study period. Secondly, circulating vitamin D level was not determined. Previous studies suggested that adipose tissue could sequester vitamin D and reduce its circulating level. Thirdly, we did not flush out the bone marrow prior to analysis, thus the role of mesenchymal stem cells on osteoblastogenesis and osteoclastogenesis should not be ruled out. Nonetheless, this is the first study investigating the role of osteocyte-related peptides in regulating bone metabolism using a MetS-induced osteoporotic rat model that was treated with tocotrienol. 

## 5. Conclusions

MetS mainly causes bone loss by increasing the levels of sRANKL, SOST, DKK-1, and FGF-23, leading to an imbalance between bone formation and resorption. Treatment with tocotrienol for 12 weeks can normalize the changes in these bone-related peptides that were caused by MetS, thus reiterating the anti-osteoporotic potential of tocotrienol. The findings from present studies support the idea that osteocytes are important coordinators for bone metabolism. Future pharmacologic approach for the prevention and treatment of osteoporosis may focus on targeting osteocytes to increase bone mass and bone strength.
